# The impact of voluntary folate fortification of corn masa flour on US pregnancies complicated by neural tube defects

**DOI:** 10.1007/s00381-023-05945-w

**Published:** 2023-04-26

**Authors:** Syed I. Khalid, Kyle Thomson, Brittany M. Hunter, Roxanna M. Garcia, Robin Bowman, Sandi Lam

**Affiliations:** 1 Department of Neurosurgery, University of Illinois at Chicago, Chicago, IL USA; 2 Chicago Medical School, Rosalind Franklin University of Medicine and Science, North Chicago, IL USA; 3 Department of Pediatric Hospital Medicine, Ann & Robert H. Lurie Children’s Hospital of Chicago, Chicago, IL USA; 4 Division of Pediatric Neurosurgery, Department of Neurological Surgery, Ann & Robert H. Lurie Children’s Hospital, Northwestern University Feinberg School of Medicine, Chicago, IL USA

**Keywords:** Neural tube defects, Pregnancy complications, FDA, Preventable complications of pregnancy

## Abstract

**Introduction:**

In 1996, the US Food and Drug Administration (FDA) mandated folic acid fortification for all enriched cereal grains. This resulted in a reduction of neural tube defect (NTD)–affected pregnancies. However, Hispanic women continued to be twice as likely to give birth to a child affected by NTD compared to non-Hispanic White women. Some hypotheses explaining this difference focus on cultural variation in dietary intake of cereal grains. In 2016, the FDA approved voluntary folic acid fortification for corn masa flour products to focus on the Hispanic diet staple. This study investigates rates of NTDs in predominantly Hispanic-populated zip codes before and after the voluntary fortification of corn masa flour with folic acid.

**Methods:**

Normal pregnancies and those complicated by NTDs between 1/1/2016 and 9/30/2020 were identified using ICD-9 and ICD-10 codes in an all-payor claims database. The post-fortification period began 12 months after the fortification recommendation. The US Census data was used to stratify pregnancies in predominantly Hispanic zip codes (≥ 75% of households) vs. non-Hispanic zip codes. The causal impact of the FDA’s recommendation was assessed by means of a Bayesian structural time series model.

**Results:**

A total of 2,584,366 pregnancies were identified among females aged 15–50 years. Of these, 365,983 took place in predominantly Hispanic zip codes. Mean quarterly NTDs per 100,000 pregnancies did not significantly differ between predominantly Hispanic zip codes and predominantly non-Hispanic zip codes pre-FDA recommendation (184.5 vs. 175.6; *p* = 0.427), nor post-recommendation (188.2 vs. 185.9; *p* = 0.713). Rates of NTDs predicted to occur if no FDA recommendation had been made were compared to the actual rate post-recommendation: no significant difference was observed in predominantly Hispanic zip codes (*p* = 0.245) or overall (*p* = 0.116).

**Conclusions:**

Rates of neural tube defects were not significantly reduced in predominantly Hispanic zip codes following the 2016 FDA approval of voluntary folic acid fortification of corn masa flour. Further research and implementation of comprehensive approaches to advocacy, policy, and public health are necessary to decrease preventable congenital disease rates. Mandatory rather than voluntary fortification of corn masa flour products may achieve more substantial prevention of neural tube defects in at-risk US populations.

**Supplementary Information:**

The online version contains supplementary material available at 10.1007/s00381-023-05945-w.

## Introduction

In 1996, the US Food and Drug Administration (FDA) mandated folic acid fortification for all enriched cereal grains by 1998 [1]. Increasing folic acid intake across the population, thus women of childbearing age, represents efforts to reduce the incidence of neural tube defects (NTDs). These congenital malformations result from failure of neural tube closure by the 28th day of gestation. Failure of neural tube closure can occur anywhere along the neural axis but is most common in the cranial or caudal spinal regions [2]. Folic acid deficiency is among several risk factors for NTDs but is positively influenced by proper prenatal nutrition. Over half of NTDs are folic acid–sensitive and can be prevented with sufficient folic acid consumption before conception and early pregnancy [3].

Following the FDA implementation of folic acid fortification in enriched cereal grains, there was a decrease in reported NTD-affected pregnancies in the USA from 4130 in 1995–1996 to 3020 in 1999–2000 [4]. Despite this large-scale success in decreasing folic acid–sensitive NTDs, Canfield et al. [6] along with the National Birth Defects Prevention Network showed that when compared to non-Hispanic White women, Hispanic women continued to be more likely to give birth to a child affected by anencephaly (adjusted prevalence ratio 1.64, 95% CI 1.47–1.83) and/or spina bifida (adjusted prevalence ratio 1.24, 95% CI 1.15–1.33) [5, 6]. There are limited data demonstrating a genetic predisposition of Hispanic populations to NTDs. Most hypotheses explaining this difference in NTD rates depend on socioeconomic and health-related disparities and cultural differences in the dietary intake of cereal grains [7]. To address the disparity, the FDA introduced voluntary fortification of corn masa flour in April 2016, commonly a food staple in the typical Hispanic diet [1, 8]. Our current study investigates rates of NTDs before and after the voluntary folic acid fortification of corn masa flour in predominantly Hispanic-populated zip codes and zip codes that are not predominantly Hispanic.

## Methods

### Data source

This study followed the Strengthening the Reporting of Observational Studies in Epidemiology reporting guidelines. Longitudinal Analytic Files containing 100% of all inpatients, outpatients, drug, and laboratory claims of 122 million patient claims from an all-payor database, MARINER, between January 1, 2016, and September 30, 2020, were analyzed [9]. The study was provided a waiver of patient informed consent by our institutional review board as the nature of this analysis posed minimal risk to participating individuals, and the data was presented in aggregate to minimize any risk of loss of confidentiality of medical data.

### Study cohort

Pregnancies and NTDs occurring among non-high-risk pregnancies of patients between 15 and 50 years of age were identified using the International Classification of Diseases, Ninth Revision (ICD-9), and International Statistical Classification of Diseases and Related Health Problems, Tenth Revision (ICD-10) diagnostic codes (Supplemental Table S1). The US Census data was used to determine zip codes where > 75% of people were of Hispanic or Latino origin. Those zip codes were then used to separate patients into two cohorts: those who received care in zip codes with a population composed of ≥ 75% Hispanic persons, and those who received care in zip codes with a population consisting of < 75% Hispanic persons (Supplemental Table S2).

**Table 1:** Descriptive characteristics for patients with pregnancies complicated by neural tube defects before and after initiation of folate fortification of corn masa flour in 2016

	**Before** NTD/total pregnancies ***n* = 3509/794,491**	**After** NTD/total pregnancies ***n* = 10,989/1,789,875**	***p*-value**
**Age** *n* (%)			0.129/ < .001*
15–19	198 (5.6)/66,144 (8.3)	604 (5.5)/171,937 (9.6)	
20–24	782 (22.3)/179,610 (22.6)	2541 (23.1)/378,327 (21.1)	
25–29	941 (26.8)/204,455 (25.7)	2975 (27.1)/432,085 (24.1)	
30–34	954 (27.2)/177,772 (22.4)	2745 (25)/385,454 (21.5)	
35–39	493 (14)/107,084 (13.5)	1642 (14.9)/253,734 (14.2)	
40–44	141 (4)/41,084 (5.2)	441 (4)/112,162 (6.3)	
45–50	0 (0)/18,342 (2.3)	41 (0.4)/56,176 (3.1)	

*Significant values *P* < 0.05

**Table 2:** Descriptive characteristics for patients with pregnancies complicated by neural tube defects by Hispanic versus non-Hispanic zip codes before initiation of folate fortification of corn masa flour

	**Non-Hispanic zip codes** NTD/total pregnancies ***n* = 3007/687,651**	**Hispanic zip code** NTD/total pregnancies ***n* = 502/106,840**	***p*-value**
**Age** *n* (%)			0.159/ < .001*
15–19	174 (5.8)/57,669 (8.4)	24 (4.8)/8475 (7.9)	
20–24	668 (22.2)/156,818 (22.8)	114 (22.7)/22,792 (21.3)	
25–29	804 (26.7)/177,512 (25.8)	137 (27.3)/26,943 (25.2)	
30–34	836 (27.8)/153,477 (22.3)	118 (23.5)/24,295 (22.7)	
35–39	409 (13.6)/91,348 (13.3)	84 (16.7)/15,736 (14.7)	
40–44	116 (3.9)/34,890 (5.1)	25 (5)/6194 (5.8)	
45–50	0 (0)/15,937 (2.3)	0 (0)/2405 (2.3)	

*Significant values *P* < 0.05

### Outcome definitions

The primary aim of this study was to assess the change in the proportion of pregnancies impacted by NTDs before and after the FDA’s voluntary folic acid fortification of corn masa flour. The post-fortification period started 1 year after the 2016 fortification, stratified by age categories.

### Statistical analysis

*χ*^2^ tests were used to compare categorical variables including age and geographic region. ANOVA tests were used for quantitative variables, like quarterly rates of NTDs. Quarterly rates of NTDs were expressed per 100,000 pregnancies among women ages 10 to 50 years. A Bayesian structural time series model was developed on the basis of NTDs per 100,000 pregnancies and total pregnancies so as to predict how the rates of NTDs per 100,000 pregnancies among women would have evolved if the FDA’s recommendation for voluntary folic acid fortification of corn masa flour had never occurred. This counterfactual inference was then compared to the actual rates of NTDs per 100,000 pregnancies as previously described [10]. No single statistical method for time series analysis has been shown to be superior; thus, the Bayesian model was selected for its interpretability. Data was analyzed using R statistical software version 4.0 (The R Foundation) (Table 1).

## Results

### Descriptive characteristics

Between January 1, 2016, and September 30, 2020, 2,584,366 total pregnancies were identified among females between the age of 15 and 50 years. Greater than 60% of pregnancies in both the pre- and post-intervention periods took place among women aged 20–34 years (Table 2). Of all identified pregnancies, 365,983 took place in zip codes with a population composed of ≥ 75% Hispanic persons. 2152 of which were found to be complicated by NTDs, 502 before and 1650 in the period after the FDA’s recommendation for the voluntary folic acid fortification of corn masa flour (Tables 2 and 3).

**Table 3:** Descriptive characteristics for patients with pregnancies complicated by neural tube defects in Hispanic versus non-Hispanic zip codes after initiation of fortification of corn masa flour

	**Non-Hispanic zip codes** **NTD/total pregnancies** ***n* = 9346/1,540,544**	**Hispanic zip code** **NTD/total pregnancies** ***n* = 1650/259,143**	***p*-value**
**Age** *n* (%)			0.024*/ < .001*
15–19	174 (5.8)/57,669 (8.4)	24 (4.8)/8475 (7.9)	
20–24	668 (22.2)/156,818 (22.8)	114 (22.7)/22,792 (21.3)	
25–29	804 (26.7)/177,512 (25.8)	137 (27.3)/26,943 (25.2)	
30–34	836 (27.8)/153,477 (22.3)	118 (23.5)/24,295 (22.7)	
35–39	409 (13.6)/91,348 (13.3)	84 (16.7)/15,736 (14.7)	
40–44	116 (3.9)/34,890 (5.1)	25 (5)/6194 (5.8)	
45–50	0 (0)/15,937 (2.3)	0 (0)/2405 (2.3)	

*Significant values *P* < 0.05

### Rates of NTDs in predominantly Hispanic zip codes vs. predominantly non-Hispanic zip codes

The mean quarterly NTDs per 100,000 pregnancies in predominantly Hispanic zip codes in the pre-intervention period was 184.5 and did not differ significantly from the quarterly mean 175.6 NTDs per 100,000 pregnancies in zip codes with < 75% Hispanic persons (*p* = 0.427). Likewise, the mean quarterly NTDs per 100,000 pregnancies did not differ between predominantly Hispanic zip codes and predominantly non-Hispanic zip codes in the post-intervention period (188.2 vs. 185.9; *p* = 0.713) (Table 4).

**Table 4:** Neural tube defects per 100,000 live births per quarter before and after (grey) voluntary folate fortification of corn masa flour

**Year, quarter**	**Zip codes with** ≥ **75% Hispanic persons**	**Zip codes with < 75% Hispanic persons**	***p*-value**
2016, Q1	156.8	174.1	
2016, Q2	201.4	179.4	
2016, Q3	186.7	171.3	
2016, Q4	193.4	177.4	
**Mean pre-intervention**	**184.5**	**175.6**	**0.427**
2017, Q1	178.7	194	
2017, Q2	173.3	181.6	
2017, Q3	191	163.1	
2017, Q4	170.7	149.7	
2018, Q1	215.5	174.4	
2018, Q2	202.5	195.6	
2018, Q3	219.2	182.8	
2018, Q4	181.8	169.6	
2019, Q1	195.3	193.7	
2019, Q2	175.9	197.9	
2019, Q3	179.8	187.8	
2019, Q4	179	186.8	
2020, Q1	188.5	196.7	
2020, Q2	211.1	217.8	
2020, Q3	160.1	196.5	
**Mean post-intervention**	**188.2**	**185.9**	**0.713**

### Impact of the FDA’s recommendation on NTDs

**Fig. 1:**
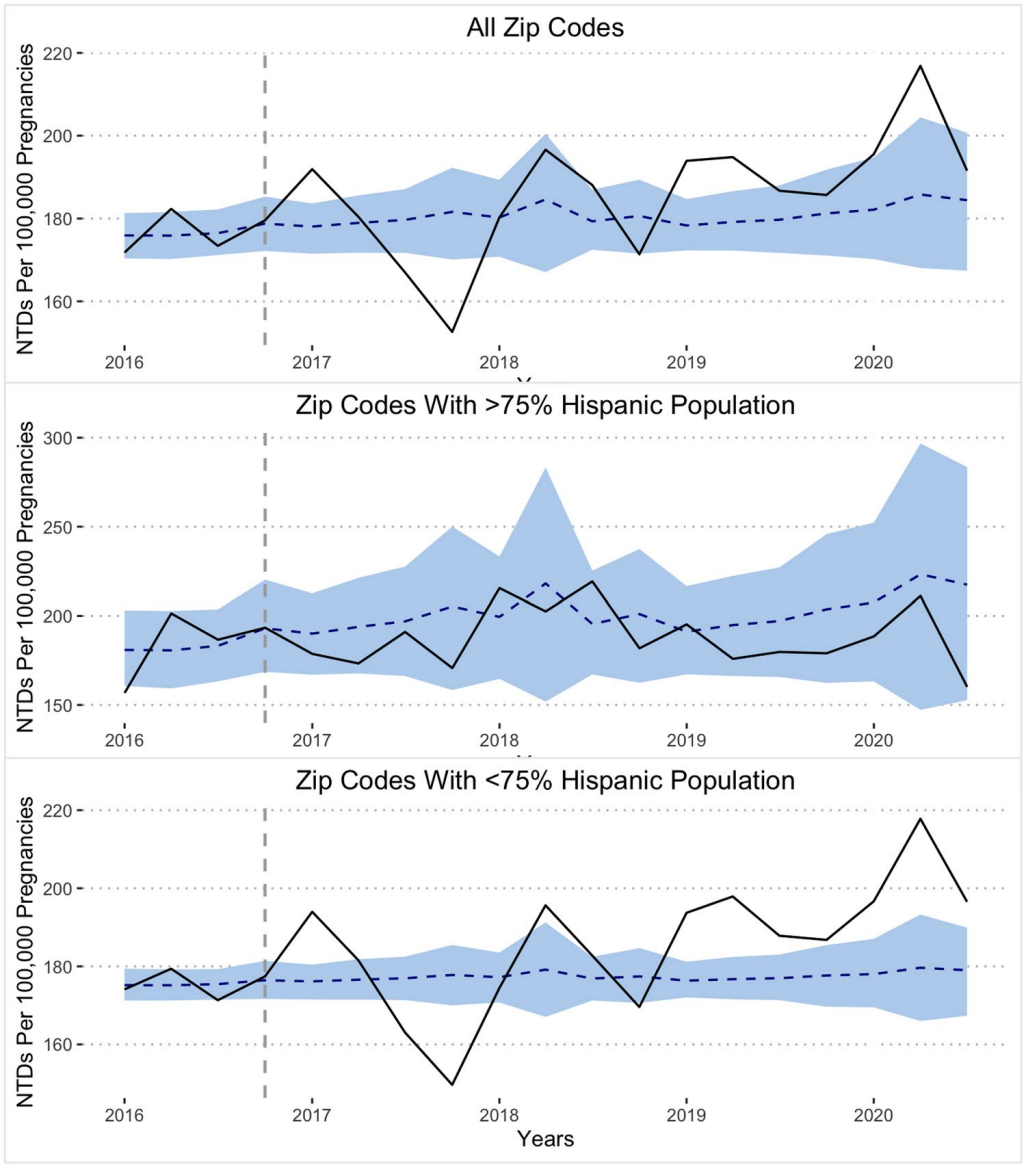
Neural tube defects per 100,000 live births per quarter before and after the voluntary folate fortification of corn masa flour. Solid black: actual number of NTDs per 100,000 live births. Blue dashed line: projected number of NTDs per 100,000 live births. Shaded blue region: confidence interval for projection. Dashed grey line: border between before and after implementation periods

Using a structural time series model, we compared the rates of neural tube defects per 100,000 pregnancies predicted to occur if no Food and Drug Administration recommendation was made to those that were observed to occur following the Food and Drug Administration’s recommendation. The predicted mean quarterly NTDs per 100,000 pregnancies for the post-intervention period across all zip codes was found to be 181 (95% CI 172–190), which is 2.9% lower than the observed. No statistically significant causal impact on rates of NTDs per 100,000 pregnancies was found as a result of the FDA’s recommendation for the voluntary fortification of corn masa flour (95% CI − 1.9% to 7.9%; *p* = 0.116). In predominantly Hispanic zip codes, the predicted mean quarterly NTDs per 100,000 pregnancies for the post-intervention period was 202, which was 7.0% higher than the observed rate (188). This difference was not significant (95% CI − 24–11%; *p* = 0.245). Finally, in predominantly non-Hispanic zip codes, the predicted mean quarterly NTDs per 100,000 pregnancies for the post-intervention period was 178, which was 4.7% less than the observed rate (186). This difference was statistically significant (95% CI 1.1–8.2%; *p* = 0.006) (Fig. 1).

## Discussion

While the 1998 FDA mandate of cereal grain fortification of folic acid resulted in the reduction of NTDs in the USA, Hispanic American women continued to be more likely to give birth to children affected by NTDs [11]. This vulnerability was at least in part attributed to the low cultural intake of enriched cereal grains, which prompted the 2016 initiative for voluntary fortification of corn masa flour, a food staple in a typical Hispanic diet in tortilla products, tamales, and more [1, 8]. Our study investigated the rate of NTDs around the time of voluntary folic acid fortification of corn masa flour. We found that in the USA, the rate of NTDs in predominantly Hispanic zip codes receiving care following the 2016 voluntary folic acid fortification of corn masa flour was not significantly different compared to the counterfactual prediction. On the other hand, following the 2016 FDA recommendation of voluntary fortification, the rate of NTDs in predominantly non-Hispanic zip codes receiving care was higher than the counterfactual prediction (Fig. 1). Notably, three of the four highest quarterly rates of NTDs came in 2020 (Table 4). The higher-than-expected rate of NTDs in zip codes that were predominantly non-Hispanic could be due to several factors such as the COVID-19 pandemic, recent changes in Western diets [12], limitations of statistical estimation of other unmeasured confounders, or type I error.

In 1998, the FDA mandated that all enriched cereal grains be fortified with folic acid. In the 22 years since its implementation, the Center for Disease Control and Prevention announced a 36% reduction in the number of NTDs between 1996 and 2006 [13]. In addition to decreasing the risk of NTD-related lifetime disability, folic acid fortification is estimated to reduce the present value of total direct costs for each year’s birth cohort by $603 million more than the cost of fortification [14].

While the fortification of enriched cereal grains with folic acid proved to be widely successful in reducing the total number of NTDs in the USA, Hispanic women benefited less than non-Hispanic women. The cultural factors affecting the dietary consumption of enriched cereal grains led to petitions for the fortification of corn masa flour, such as that found in tortilla products [15]. Hamner et al. [15] predicted that this step could selectively increase the total folic acid intake among Mexican American women, especially those preserving more of their Mexican cultural and dietary identity. As such, this public health intervention targets Hispanic women who consume corn masa flour, which may not apply to all culturally divergent dietary patterns of Hispanic women living in the USA or captured by these data. Latin American countries had begun to see results of dietary fortification much earlier following fortification efforts. Lopez-Camelo et al. [16] analyzed if the rate of NTDs was decreasing due to folic acid fortification of wheat flour or preexisting trends in Chile between 1982 and 2002, following the introduction of fortification in 2000. They determined there was a 43% reduction in the rate of NTDs during mandatory fortification efforts [16].

Our study demonstrates that the USA’s introduction of voluntary corn masa folic acid fortification does not correspond temporally with decreased rates of NTDs in live births in predominantly Hispanic zip codes receiving care. Twenty months following the 2016 initiative, a survey of grocery stores in Atlanta, GA, determined that only 10% of corn masa flour products and none of the soft corn tortilla products were fortified with folic acid [17]. Another study confirmed rates as low as 7% of products were fortified [18]. Likewise, our results demonstrate no temporal relationship of decreased rates of NTDs among pregnancies following voluntary fortification of corn masa flour products. Other national databases have shown a similar impact of voluntary fortification efforts in the USA [19]. Using the National Health and Nutrition Examination Survey (NHANES) data, Wang et al. [19] demonstrated that RBC folate concentrations in Hispanic women of reproductive age did not differ after voluntary fortification of corn masa flour products (2017–2018) compared to before (2011–2016). It is difficult to surmise the uptake of the intervention based on these data given its voluntary nature. Furthermore, it has been shown that mandatory fortification is more effective than recommended fortification to reach large portions of the population, thereby achieving the desired health impact [20]. It is plausible that mandatory fortification of corn masa flour products would be necessary to reach those sub-populations of Hispanic women who are at the most significant risk for pregnancies complicated by NTDs.

### Limitations

This study is not without limitations. Like all observational studies, this study is prone to unobserved confounding and selection bias. The current study captured a relatively short time period compared to other epidemiological studies demonstrating population effects [4, 16], and may require a longer time frame to demonstrate the uptake of fortification intervention and its impact among the target population. The interrupted time series analysis examines the effects of an intervention or policy change on the observed outcomes. We are unable to account for the changing US demographics with possible dietary acculturation in women of childbearing age. Confounding health conditions in this population, if present, is not measured directly. The Mariner Database does not include patient race, surgeon characteristics, or other granular data such as adherence to prenatal care, planned pregnancy status, or other clinical decision-making.

Nevertheless, it serves as a valuable evaluation among the 122 million patient database. Notably, uninsured, illegal, or immigrant Hispanic women may not have been captured and may be at the most significant risk for NTDs during pregnancy. These data are difficult to obtain and should be prioritized among public health efforts for reproductively active Hispanic women in the USA.

## Conclusion

Our current study demonstrates no significant decrease in rates of NTDs in pregnancies following voluntary corn masa folic acid fortification. Compared to the 1998 FDA mandate of folic acid fortification of enriched cereal grains, which was followed by a significant reduction in NTD rates, the 2016 FDA announcement of voluntary fortification of corn masa flour products was not followed by a decrease in NTD rates in the Hispanic population. Neural tube defects represent preventable pregnancy complications and carry high morbidity and mortality. Multimodal advocacy and policy can have a significant benefit for future generations. Mandatory rather than voluntary fortification of corn masa flour products may achieve more substantial prevention of neural tube defects in at-risk US populations.

## Supplementary Information

Below is the link to the electronic supplementary material.

Supplementary file1 (DOCX 15 KB)

## Data Availability

The data used in this study are publicly available for purchase.
