# Supplementary material for: The impact of voluntary folate fortification of corn masa flour on US pregnancies complicated by neural tube defects
Source: Childs Nerv Syst. 2023 Apr 26;39(7):1813–9. doi: 10.1007/s00381-023-05945-w (PMC10132414; doi:10.1007/s00381-023-05945-w)
Supplement: Supplementary file 1 — Supplementary file1 (DOCX 15 KB) [file 381_2023_5945_MOESM1_ESM.docx]

***Solid black:*** *actual number of NTDs per 100,000 live births.*

***Blue dashed line****: projected number of NTDs per 100,000 live births.*

***Shaded blue region:*** *confidence interval for projection. Dashed grey line: border between before and after implementation periods.*

|  | **Average**  (SD) [95% CI] | **Cumulative**  (SD) [95% CI] | **p-value** |
| --- | --- | --- | --- |
|  | **All Zip Codes** | |  |
| **Actual** *n* | 186 | 2793 |  |
| **Prediction** *n* | 181 (4.4) [172, 190] | 2714 (66.2) [2580, 2845] |  |
| **Absolute Effect** *n* | 5.3 (4.4) [-3.5, 14] | 79.6 (66.2) [-51.8, 213] |  |
| **Relative Effect** *n* *(%)* | 2.9% (2.4%) [-1.9%, 7.9%] | 2.9% (2.4%) [-1.9%, 7.9%] | .116 |
|  | **Zip Codes With** ≥**75% Hispanic Population** | |  |
| **Actual** *n* | 188 | 2823 |  |
| **Prediction** *n* | 202 (17) [166, 237] | 3035 (260) [2495, 3555] |  |
| **Absolute Effect** *n* | -14 (17) [-49, 22] | -213 (260) [-732, 327] |  |
| **Relative Effect** *n* *(%)* | -7% (8.6%) [-24%, 11%] | -7% (8.6%) [-24%, 11%] | .245 |
|  | **Zip Codes With <75% Hispanic Population** | |  |
| **Actual** *n* | 186 | 2788 |  |
| **Prediction** *n* | 178 (3.2) [171, 184] | 2664 (47.4) [2570, 2759] |  |
| **Absolute Effect** *n* | 8.3 (3.2) [1.9, 15] | 123.9 (47.4) [29.0, 218] |  |
| **Relative Effect** *n* *(%)* | 4.7% (1.8%) [1.1%, 8.2%] | 4.7% (1.8%) [1.1%, 8.2%] | .006* |
| ****Significant Values P <0.05*** |  |  |  |

**Supplemental Table 1.** International Classifications of Disease (ICD) – 9 and –10 Diagnosis Codes Utilized to Access Births Complicated by Neural Tube Defects

| **Category** | **Billing Code** |
| --- | --- |
| Neural Tube Defect | **ICD-9:** 655.00, 655.01, 655.03  **ICD-10:** O350XX0 |
| Pregnancy | **ICD-9:** V220, V221  **ICD-10:** Z3400, Z3480, Z3490, Z3401, Z3402, Z3403, Z3481, Z3482, Z3483, Z3491, Z3492, Z3493 |

**Supplemental Table 2.** Zip Codes Reported as ≥75% Hispanic by United States of America Census Data

| **Zip Codes** |
| --- |
| 95937,93627,78595,78547,85349,78576,78579,79853,78548,93234,88027,78562,79849,88063,78584,78557,78558,78582,78543,78560,92236,90023,92249,78046,90063,78592,78565,78040,78860,90270,90022,79905,87736,85336,78349,90255,78211,78588,79901,78852,93249,79838,78589,78043,79780,92231,93608,78549,78225,77011,92254,79907,78371,78237,78353,78359,79013,88024,78538,79915,93640,78521,78570,78594,79845,90040,33135,78827,93648,78839,78207,90201,78593,95365,87583,79835,79927,93239,85621,90033,90280,78224,88048,77012,93261,33010,78041,78503,87515,78384,87723,78204,87537,93615,33126,78355,78369,78577,87516,78376,88072,93660,33130,33013,87017,87511,33174,78045,78563,78361,33012,33125,78569,79903,78520,78539,85329,87711,78526,78405,93624,78214,78830,78586,93241,87578,93905,92173,33128,33144,87724,78829,78537,33018,91340,87715,79836,92701,81153,78572,88022,90660,78596,87522,92274,33184,79839,87732,93646,78339,33016,78417,78145,81126,79930,78344,85714,90304,79846,87512,87569,87567,87582,87012,78019,87521,87060,93622,77023,78580,93666,90017,88353,79821,33172,93926,78416,93219,90011,78228,78516,78872,87530,33145,87941,78076,90744,93927,90001,93434,93606,33175,79938,93203,78501,87064,93960,78834,87519,78385,92318,87553,78221,78351,87560,88021,78566,88023,87713,88026,76106,91331,93250,87548,91746,90262,78067,78352,90606,78201,78061,92273,87701,87581,87575,85235,79936,33165,91744,81152,78375,95653,87543,87524,90015,78210,92281,87937,88044,90032,85034,88435,91733,78242,87579,81124,87936,85648,11237,78332,78836,80216,92703,81120,92707,79718,95387,93647,85607,78583,87562,91706,60165,87940,90006,93954,33014,33185,77587,78357,78380,90058,60804,88029,93616,75220,87753,85350,78014,75211,87121,95951,33193,87105,87041,78226,33182,85009,87532,79902,78559,10040,78840,93458,01107,78011,33155,93235,87549,93665,95002,07087,95012,95019,79719,87552,78415 |
